# Evidence for the ‘Good Genes’ Model: Association of MHC Class II *DRB* Alleles with Ectoparasitism and Reproductive State in the Neotropical Lesser Bulldog Bat, *Noctilio albiventris*

**DOI:** 10.1371/journal.pone.0037101

**Published:** 2012-05-16

**Authors:** Julia Schad, Dina K. N. Dechmann, Christian C. Voigt, Simone Sommer

**Affiliations:** 1 Leibniz Institute for Zoo and Wildlife Research, Evolutionary Genetics, Berlin, Germany; 2 Department of Migration and Immuno-Ecology, Max Planck Institute for Ornithology, Radolfzell, Germany; 3 Department of Biology, Universität Konstanz, Konstanz, Germany; 4 Leibniz Institute for Zoo and Wildlife Research, Evolutionary Ecology, Berlin, Germany; Duke-NUS, Singapore

## Abstract

The adaptive immune system has a major impact on parasite resistance and life history strategies. Immunological defence is costly both in terms of immediate activation and long-term maintenance. The ‘good genes’ model predicts that males with genotypes that promote a good disease resistance have the ability to allocate more resources to reproductive effort which favours the transmission of good alleles into future generations. Our study shows a correlation between immune gene constitution (Major Histocompatibility Complex, MHC class II *DRB*), ectoparasite loads (ticks and bat flies) and the reproductive state in a neotropical bat, *Noctilio albiventris*. Infestation rates with ectoparasites were linked to specific *Noal-DRB* alleles, differed among roosts, increased with body size and co-varied with reproductive state particularly in males. Non-reproductive adult males were more infested with ectoparasites than reproductively active males, and they had more often an allele (Noal-DRB*02) associated with a higher tick infestation than reproductively active males or subadults. We conclude that the individual immune gene constitution affects ectoparasite susceptibility, and contributes to fitness relevant trade-offs in male *N. albiventris* as suggested by the ‘good genes’ model.

## Introduction

The possible involvement of immune function in trade-offs with life-history related traits is increasingly being recognized as an important aspect of life-history evolution. In particular, it is expected that competitive allocation of resources occurs between reproductive effort and immunocompetence [1]–[3]. In vertebrates, trade-offs between costs (reproductive investment or immunocompetence) and benefits (current or future reproductive success) have to be mediated in both sexes. Investment in reproductive effort may lead to suppressed immune function with the consequence of an increased susceptibility to parasites [4], [5]. But evidence has also been reported for a reverse interaction with immune activation restraining reproductive investment especially in males as an effect of reduced testosterone levels [6]–[8]. According to the for males developed ‘good genes’ model, the immune response of individuals with a well adapted immune system to parasites should be less costly leaving more resources to other fitness enhancing traits. Consequently, males with ‘good genes’ for parasite resistance may tolerate the high costs of reproduction better, leading ultimately to an increased fitness and to a spread of these immune genes into subsequent generations [1], [4], [9].

The most important immune genes in the context of parasite resistance and reproduction are those found in the major histocompatibility complex (MHC; reviewed e.g. [10]). Genes within the MHC are involved in the adaptive immune response and are among the most variable genes in vertebrates [11]. This polymorphism enables the immune system to recognize an extensive range of extra- (e.g. bacteria, helminths, arthropods via MHC class II genes) and intracellular (e.g. viruses, cancer cells via MHC class I genes) pathogens and is crucial for the immunological fitness within an individual and across animal populations [12], [13]. Haematophageous ectoparasites induce host immune regulatory and effector pathways, which involve antibodies, complement and cytokines of the innate immune system, as well as antigen-presenting cells and T-lymphocytes of the adaptive immune pathway [14], [15]. Antigens derived from anticoagulants, antiplatelets, vasodilators and immunmodulaters, which are present in the saliva of ectoparasite arthropods to evade host haemostatic defences, are processed and presented to antigen-specific T-lymphocytes at ectoparasite attachment sites by specific host MHC class II molecules. Subsequently, T-lymphocytes provide immunoregulatory signals for the production of cell-mediated antibody responses which impair the ability of a constant blood flow throughout the blood meal by inactivating saliva mediated proteins. Acquired resistance to ectoparasite infestation may lead to a reduced feeding time, affects number and viability of ova, and may even cause death of ticks during feeding [14], [16]. On the other side, for both rapidly feeding insects and slowly feeding ticks the reduction of host immunity to their salivary components enhances the likelihood that a host will be a suitable source of future blood meals driving a co-evolutionary arms race [15], [17]–[19]. Furthermore, in the host immunological mediators contribute to an itch sensation, which stimulates self-grooming [20], [21], an important factor in reducing ectoparasite burden [22]. Thus, immunologically acquired host resistance to ectoparasite feeding may decrease ectoparasite infestation intensity [14], [15], [19]. However, immunological defence to haematophagous ectoparasites is costly both in terms of activation and maintenance and is therefore subject to trade-offs among an organism's competing energy requirements [21]–[23].

In addition to the immunological and behavioural defences, ectoparasite abundance is also influenced by environmental factors (temperature and humidity) and host characteristics such as home range, social system, sex, reproductive state, age and body size [23]–[26]. The relevance of these factors in determining ectoparasite abundance is likely to be specific for each host-parasite system [27]. Bat ectoparasites spend their entire lives either on the body or in the roosts of their hosts. Thus, for most bat ectoparasites, contact between host individuals is required for host transfer or is restricted to host individuals that inhabit the same roost [27], [28]. Whereas some ectoparasites may infest different bat species, some show high host specificity, indicating co-evolutionary adaptation processes [29], [30]. Bats provide a favourable opportunity to study effects and adaptive processes between ectoparasites and host immune defence, especially with regard to host's MHC genes.

In a previous study we investigated MHC class II polymorphism in a natural population of the lesser bulldog bat, *Noctilio albiventris*, in Panama. The single expressed highly variable MHC class II *Noal-DRB* locus showed clear signs of selection shaping the diversity pattern [31]. The population is infested by two main haematophaegeous ectoparasites, the tick *Ornithodoros hasei* (Argasidae), which is known to infest also other bat species and the host-specific bat fly *Paradyschiria parvuloides* (Streblidae) (Dechmann, personal observation, [32]). *Noctilio albiventris* lives in social groups year-round, and these social groups consist commonly of several females and non-reproductive as well as reproductive males (Dechmann, personal observation). Reproductive and non-reproductive adult males are observed throughout the year, which suggests that not all adult males in a population are reproductively active at the same time [33]. However, the underlying ecological and physiological causes and mechanisms have not been investigated so far. Together these make *N. albiventris* an ideal candidate to investigate the interaction between immune genes, ectoparasite susceptibility and reproductive state.

In this study, we recorded the ectoparasite loads (ticks and bat flies), the reproductive state and MHC class II *DRB* gene variability in several roosts of free-ranging *N. albiventris* and tested predictions of the ‘good genes’ model. According to the ‘good genes’ model we expected males with good genes (i.e. the *Noal-DRB* alleles) to have lower parasite loads, allowing them to invest more resources in reproduction. Thus, *Noal-DRB* alleles with a protective effect on ectoparasite burden should be more frequent in reproductive individuals, whereas *Noal-DRB* alleles that associate with high ectoparasite burden should accumulate in non reproductive individuals.

## Methods

### Ethics Statement

All capture and handling of animals as well as collection and export of samples was done in concordance with Panamanian laws. Permits were issued from the Panamanian authority Autoridad National del Ambiente (ANAM, SE/A 98-08, SEX/A78-08, SEX/A -138-08) and field work and animal handling was carried out according to the protocol by the Smithsonian Tropical Research Institute – Institutional Animal Care and Use Committee (STRI-IUCAC).

### Study site and sampling

From February to June, and September to November of the years 2006–2008, we captured 214 individuals of the lesser bulldog bat, *N. albiventris* in the village Gamboa (09.07°N, 079.41°W) and on Barro Colorado Island (BCI, 09.10°N, 079.51°W). Both sites are located at the Panama Canal. Bats were captured with mist nets when they emerged at dusk from one of six investigated roosts in Gamboa or when foraging over the Panama Canal along boat docks on BCI (see [31]). Age class (adult or subadult) was distinguished by illuminating the surface of the extended wing and examining the epiphysal-diaphyseal fusion of the fourth metacarpal-phalangeal joint which is a highly reliable method to qualitatively distinguish between these age categories. Those with open joints were classified as ‘subadults’ and those with fused joints as ‘adults’ [34]. Bats were sexed and reproductive condition of females was determined by abdominal palpation and by examination of teats as advised by Racey [35]. They were categorized as ‘pregnant’ when a foetus was detectable (this condition was probably only recognized when the gestation period was about half over), as ‘lactating’ when milk could be expressed from the nipples and as ‘non-reproductive’ when neither was observed [35]. Reproductive status in males is usually evaluated by externally visible changes in testicular and epididymal size, which are thought to signal reproductive readiness [33], [35]. In *N. albiventris* testes are temporarily enlarged and thought to indicate reproductive readiness. Simultaneously important secondary sexual trait was considered. Glandular cells in inguinal pockets of the scrotum, visible only when testes are enlarged, produce a male specific odour, which is used most probably for sexual displays [32], [36]. Accordingly, males were considered as ‘reproductively’ active when testes were distended and inguinal pockets visible. All other adult males were categorized as ‘non-reproductive’ [35].

Body mass of bats was measured by using a handheld balance (accuracy ±0.5 g). Body mass does not correlate linearly with body surface area, which is the measure of interest when analyzing ectoparasite abundance. Body surface area of small mammal species can be estimated by scaling the body mass to the power of 2/3 [37], [38]. Hence, we used bodymass^2/3^ as a proxy of body surface area to quantify linear relationships between ectoparasite abundance and host body size in our analyses (see [27], [39]). From all bats, we collected a 4-mm skin sample from the wing membrane using a sterile biopsy punch [40]. Skin samples were stored in 96% ethanol until DNA isolation.

### Parasite Screening

Direct counts of large ectoparasites (bat flies, ticks) were conducted for each captured *N. albiventris*. Bat flies were counted visually by removal from the bat. In order to minimize handling time, we could not verify minute diagnostic characters on every ectoparasite specimen counted. Thus, we recorded numbers of bat flies per bat. Ticks almost exclusively occurred on the naked surfaces of the wing and tail membrane and reached extremely high numbers. Again to minimize handling time counts were restricted to a representative area, the upper surface of the dorsal uropatagium. Voucher specimens of ectoparasites were collected opportunistically and stored in 70% ethanol. Ectoparasites were identified using dichotomous keys [41]–[43], and voucher samples were verified by L. Durden (ticks: specimens at U.S. National Tick Collection, accession numbers RML 12510–RL12513) and by C.W. Dick (bat flies: specimens at the collection with C.W. Dick).

### Molecular techniques

The molecular techniques to investigate MHC class II *DRB* variability have been described in detail elsewhere [31]. Briefly, we extracted DNA from tissue sample using DNeasy Tissue Kit (Qiagen, Hilden, Germany) following the manufacturer's protocol. We used primers *JSi1N*-*DRB* and *JSi2N*-*DRB* which amplify the whole 270 bp MHC *DRB* class II exon 2 and partial introns. Amplicons were genotyped by single strand confirmation polymorphism (SSCP) on a polyacrylamid gel. For allele identification SSCP bands were subsequently cut out of the gel and re-amplified prior to cycle sequencing analyses. Cycle sequencing was performed with an Applied Biosystems automated sequencer model 3130, using a dye terminator sequencing kit (Applied Biosystems, Forster City, CA). No more than two alleles per individual were detected which was proven by RNA analyses confirming the presence of a single MHC class II *DRB* locus in *N. albiventris*. To affirm the individual SSCP pattern each individual was screened with a second primer pair (*JSi1N2*-*DRB* and *JSi2A*-*DRB*) and alleles were verified by direct sequencing. All nucleotide sequences have been submitted to GenBank (accession numbers: HM347941–HM347958).

### Statistical analyses

Arlequin 3.0 [44] was used to calculate allele frequencies and pairwise *F*_ST_ based on haplotype frequencies (10,000 permutations) to infer population subdivision. Given that F_ST_ values can underestimate the differentiation between populations with highly polymorphic loci we also estimated the degree of differentiation using both Hedrick's *G′*_ST_ [45] and Jost's D_est_ [46] with the program Smogd 2.6 [47]. Chi-square tests were used to compare the number of alleles between groups. Alleles of homozygote individuals were counted only once. All calculations were two-tailed with a significance level at α = 0.05 and performed using Spss 16.0 (Spss Inc., Chicago, IL, USA). A post-hoc power analysis (1-ß err prob) was run to adjust for sample sizes with the program G*power 2.0 [48]. Also post-hoc tests were two tailed with α = 0.05 and the effect size index was set to w = 0.5 (according to Cohen's effect size conventions between groups, [49]).

To test the influence of different host characteristics on the ectoparasite infestation, we applied different modelling approaches. Models offer the possibility to obtain a more complete perspective on the relevance of single factors in explaining the variation in a dependant variable, when confounding effects of other variables are included. In all models, we took infestation intensity of ticks and bat flies (number of parasites per bat examined) as response variables. The error structure of both response variables (‘ticks’, ‘bat flies’) was not normally distributed, so we used generalized linear models. Using ‘raw’ count data rather than transforming them has been strongly advised by O'Hara and Kotze [50]. The models were fitted to a quasi-Poisson error structure with a log-link function. We tested the effect of the immune gene on parasite load using MHC *Noal-DRB* alleles as presence-absence-covariates and considered also the status of heterozygosity. Further, we tested the influence of ecological host characteristics on ectoparasite infestation by including specific ‘roosts’ (nominal, 6 categories) and covariates associated with life history traits, ‘reproductive state’ (nominal, 5 categories), ‘sex’ (nominal, 2 categories), age (nominal, 2 categories) and ‘body size’ (continuous) into the models. ‘Month’ (nominal, 5 categories) as well as ‘year’ (nominal, 2 (for ticks, not counted in 2006) or 3 (for bat flies) categories) were included to test for a seasonal component.

Prior to model analyses we carried out data exploration to identify outliers and to ascertain collinearity among explanatory variables [51]. Consequently, the collinear covariates ‘sex’ and ‘reproductive state’ were analysed in independent models. Also the influence of *Noal*-*DRB* alleles and heterozygosity were tested independently. In addition to Spearman rank correlations, we used variance inflation factors (VIF) to assess the extent of any remaining collinearity in nominal covariates [51], [52] using a stringent cut-off value of ≤1.5 for the VIFs [53]. Some categories of the nominal covariate ‘month’ as well as ‘year’ had a VIF >5, which might be an effect of a biased sampling. Therefore we included a random effect of ‘month’ and ‘year’ in our generalized linear mixed models (GLMM, [54]) and used Laplace approximation. Laplace approximation approximates the true GLMM likelihood rather than a quasi-likelihood, allowing the use of likelihood-based-inference (for details see [55]). We applied quasi-AIC (QAIC, ΔQAIC) for random effect and fixed effect model selection. QAIC is similar to AIC, except that the log likelihood is divided by the estimated overdispersion scale parameter of the full model [55]. QAIC and ΔQAIC offer the possibility of multiple model comparisons. Influential *Noal-DRB* alleles were first revealed by separate GLMM models in order to reduce the number of explanatory covariates. *Noal-DRB* alleles with a reasonable impact (ΔQAIC<2) on the ectoparasite infestation were subsequently tested together with the ecological host characteristics.

In parallel, we estimated generalized estimation equations (GEE, [56]) using ‘roost’ as grouping factor to overcome the likely auto-correlation of animals using the same roost. GEEs include an additional variance component to accommodate correlated data and to allow for differences among clusters. GEEs are semi-parametric because estimates rely on parametric assumptions regarding the mean and variance/covariance. We used the compound-symmetric correlation structure, assuming no specific order between the observations of the same cluster (i.e. roost), while assuming observations from different clusters to be independent. We did model selection starting with the full model and dropping each variable in turn, applying an ANOVA analysis (Wald-test) as implemented in the “geepack” package of R [56] and removed the last significant variable.

Model validation was verified by checking for normal distribution of the residuals and by plotting standardized Pearson residuals versus fitted values in GLMM and GEEs. To ensure that any extreme effects did not overtly bias the models, models were refitted with these observations removed. In addition, missing data in the predictor “body size” reduced the number of included cases in models to 122 (ticks) and 141 (bat flies) when ecological host characteristics were tested. Limitations in sample size per category combination precluded the use of interaction terms.

To summarize, we analyzed our data by two independent approaches (GLMM and GEE) with different correlation structures as the reliability of inferences can be ascertained when estimated parameters show the same tendency in different model approximations. In addition, handling overdispersed count data is statistically difficult and only few suitable approaches are available for such data. Thus, using different approximations offers several advantages. In our case, GLMMs provide the possibility of multiple model comparison through the use of QAIC and ΔQAIC. GEEs, on the other hand, have the advantage of offering significance values for estimated parameters of categories in nominal covariates with more than two levels. All statistical analyses were conducted in the software program *R* 2.11.0 [57].

## Results

### Capture and sampling success

In total, we captured 214 *N. albiventris* (91 males, 123 females) of which 20 were subadults. All 214 bats were genotyped for MHC [31]. We collected samples from 29 bats on BCI and 185 in Gamboa. In Gamboa, we captured bats from six roosts: A (N = 52), B (N = 74), C (N = 27), D (N = 20), E (N = 4) and F (N = 7). All bats on BCI were captured in nets during foraging and cannot be assigned to specific roosts (electronic supplemental material Table S1). Reproductive and non-reproductive adult males were captured throughout the year, whereas females had two pregnancy peaks per year with a main parturition in April/May and a second, smaller one at the end of the year. These peaks were followed by an increase in lactating females (Table S2).

### Ectoparasite load

The numbers of bats examined for ticks within different roosts were as follows: A (2), B (61), C (24), D (10), E (4), F (7) and on BCI (26) (Table S1). We identified only larval stages of the tick *O. hasei* on *N. albiventris*. Seventy-four percent of the examined individuals (N = 134) were infested with ticks. The number of ticks on the uropatagium per investigated individual averaged 10.4±1.1 (range: 0–55; median: 5.0).

The numbers of bats examined for bat flies within different roosts were as follows: A (19), B (73), C (27), D (20), E (4), F (7) and on BCI (16) (Table S1). Eighty-two percent of the examined bats (N = 166) were infested with ectoparasitic flies. The number of bat flies per investigated individual averaged 8.2±0.7 (range: 0–39; median: 6.0). In addition to the bat fly *P. parvuloides* which was identified from every individual that had bat flies, and was present in all roosts, we found occasionally a second streblide bat fly. *Noctiliostrebla aitkeni*, co-infesting individuals always together with *P. parvuloides*. It was missing in bats captured from roost D, E and F.

### Host characteristics influencing the ectoparasite load

#### Ticks

Both model approximations (GLMM and GEE) gave similar results and led to the same biological conclusions: The extent of a bat's infestation with ticks was influenced by the bat's specific MHC class II *DRB* alleles as well as by ecological characteristics such as roost membership, reproductive state and body size.

All GLMM models analysing the impact of MHC *Noal-DRB* alleles independent of ecological host characteristics with ΔQAIC<2 included the alleles Noal-DRB*02 and Noal-DRB*11 together with the alleles Noal-DRB*01, Noal-DRB*04, Noal-DRB*10 in different combinations. Alleles Noal-DRB*02, Noal-DRB*04 and Noal-DRB*11 were associated with a higher tick infestation, and alleles Noal-DRB*01 and Noal-DRB*10 were associated with a lower tick infestation (Table 1).

**Table 1:** MHC class II *DRB* alleles influencing ectoparasite infestation in *N. albiventris*.

A. Ticks	Factors	Estimates ± SE	t-value
Best Model	Intercept	1.989±3.682	0.54
QAIC = 183.9	Noal-DRB*02	0.342±0.388	0.88
	Noal-DRB*11	0.628±0.628	1.00
Models with	Intercept	1.992±3.647	0.55
ΔQAIC<2.0	Noal-DRB*01	−0.266±0.328	−0.31
	Noal-DRB*02	0.336±0.387	0.87
	Noal-DRB*04	0.237±0.439	0.54
	Noal-DRB*10	−0.132±0.393	−0.34
	Noal-DRB*11	0.634±0.621	1.02

B. Bat flies	Factors	Estimates ± SE	t-value
Best Model	Intercept	1.983±1.015	1.95
QAIC = 180.5	Noal-DRB*05	−0.415±0.604	−0.69
	Noal-DRB*09	0.309±0.377	0.82
	Noal-DRB*11	0.595±0.511	1.17
Models with	Intercept	1.661±1.737	0.96
ΔQAIC<2.0	Noal-DRB*01	−0.362±0.716	−0.50
	Noal-DRB*04	−0.235±0.340	0.69
	Noal-DRB*05	−0.362±0.716	−0.50
	Noal-DRB*09	0.262±0.374	0.70
	Noal-DRB*10	−0.228±0.286	−0.80
	Noal-DRB*11	0.609±0.501	1.22

Estimated regression parameters of specific *Noal-DRB* alleles influencing tick (A, N = 131) and bat flies (B, N = 165) infestation validated by GLMM models with ΔQAIC<2 (Laplace approximation with month in years as random effects). Best model and, for simplicity, averaged parameters of models with ΔQAIC<2 are shown.

GLMM: generalized linear mixed model; QAIC: quasi Akaike information criterion where the log likelihood is divided by the estimated overdispersion scale parameter of the full model; t-value: estimated parameter divided by its standard error, indicates the likelihood that the estimated parameter is not zero.

Both the GLMM and GEE model approximations that combined these five *Noal-DRB* alleles with ecological host characteristics validated ‘roost’, ‘body size’, ‘reproductive state’ and the five *Noal-DRB* alleles to be influential for individual tick infestation (Table 2). GLMM and GEE models including the five *Noal-DRB* alleles explained significantly more of the variation in the tick infestation than a model without these alleles (GLMM without alleles raised ΔQAIC to 4.3; GEE ANOVA: χ^2^ = 36.1, df = 5, p<0.001) indicating that these MHC alleles explained a determinant part of the variation in parasite loads. In models where *Noal-DRB* alleles were replaced by the variable ‘heterozygosity’, heterozygosity was identified to have no influence on the individual tick load in GEEs (χ^2^ = 2.05, df = 1, p = 0.15), but heterozygosity was validated to be associated with increased tick load in GLMMs (GLMM without ‘heterozygosity’: ΔQAIC = 5.01). Of the ecological host characteristics, roost explained a substantial part of the variation in tick infestation (GLMM without ‘roost’: ΔQAIC = 39.3; GEE: χ^2^ = 84.3, df = 5, p<0.001). Bats from roost D and F showed a significantly higher infestation rate than animals from BCI (Table 2). In addition, body size (GLMM without ‘body size’: ΔQAIC = 33.4; GEE: χ^2^ = 41.4, df = 1, p<0.001) and reproductive state (GLMM without ‘reproductive state’: ΔQAIC = 18.8; GEE: χ^2^ = 28.2, df = 4, p<0.001) had an effect on the infestation, with non-reproductive adult males showing a significantly higher infestation rate than reproductive males. Non-reproductive adult females did not differ in their infestation rate compared to lactating and pregnant females (Table 2). Bats captured in 2008 were significantly less infected than individuals sampled in 2007 (Table 2). Estimated parameters of the optimal GLMM model revealed the same tendency as in the optimal GEE model, with increased divergences in estimates for different roosts (Table 2).

**Table 2:** Tick infestation in *N. albiventris*.

GLMM			GEE			
Factors	Estimates ± SE	t-value	Factors	Estimates ± SE	Wald-Test	p-value
Intercept	−5.580±3.560	−1.57	Intercept	−4.182±1.156	13.10	<0.001***
Roost B^1^	0.914±0.748	1.22	Roost B^1^	0.723±0.371	3.78	0.052(*)
Roost C^1^	1.188±0.783	1.52	Roost C^1^	0.569±0.440	1.67	0.196
Roost D^1^	2.043±1.253	1.63	Roost D^1^	2.398±0.401	35.73	<0.001***
Roost E^1^	−0.208±1.350	−0.15	Roost E^1^	−0.264±0.598	0.20	0.659
Roost F^1^	3.761±1.600	2.35	Roost F^1^	1.423±0.454	9.79	0.002**
Body size	0.664±0.192	2.89	Body size	0.630±0.098	41.42	<0.001***
Female lactating^2^	0.163±0.482	0.34	Female lactating^2^	0.168±0.202	0.68	0.408
Female pregnant^2^	−0.345±0.919	−0.36	Female pregnant^2^	−0.569±0.483	1.39	0.566
Male non-reproductive^3^	1.300±0.671	1.94	Male non-reproductive^3^	1.286±0.354	13.21	<0.001***
Noal-DRB*01	−0.392±0.733	−0.53	Noal-DRB*10	−0.311±0.142	4.80	0.029*
Noal-DRB*02	0.160±0.312	0.51	Noal-DRB*11	0.543±0.204	7.07	0.007**
Noal-DRB*04	0.132±0.349	0.38	Capture year 2008	−1.257±0.249	25.54	<0.001***
Noal-DRB*10	−0.236±0.302	−0.78				
Noal-DRB*11	0.516±0.511	1.01				

Estimated regression parameters and standard errors of combined ecological host characteristics and specific MHC class II *DRB* alleles on tick infestation obtained by GLMM (Laplace approximation with month and year as random effects) and GEE (with autocorrelation factor of roost: correlation parameter α = 0.03±0.07, overdispersion scale parameter = 7.1). Averaged estimates of GLMM models with ΔQAIC<2 are shown (QAIC_best_ = 182.9, overdispersion parameter of the full GLMM model = 4.57). N = 122.

1 compared to animals of BCI,

2 compared to non-reproductive adult females,

3 compared to reproductively active males.

In models where reproductive state was replaced by the variable ‘sex’, the results differed in the two model approaches. Whereas sex was validated as not influencing individual tick load in GEEs (χ^2^ = 1.18, df = 1, p = 0.28), GLMMs validated males to be less infested compared to females. However a GLMM model excluding the variable ‘sex’ explained the infestation also reasonable well (GLMM without ‘sex’: ΔQAIC = 2.94) indicating a minor effect of sex on tick loads. The association of age with individual tick load revealed heterogeneous results, which might be the effect of low sampling of subadults. In GEEs age was identified to be influential with subadults being more infested (χ^2^ = 5.35, df = 1, p = 0.02). GLMMs validated the variable ‘age’ to have a minor effect on infestation, though a model without the variable ‘age’ explained tick load best, a model including age was validated still to be realistic (GLMM with ‘age’: ΔQAIC = 1.32). Estimates of all other covariates suggested a similar influence independently whether or not the variable ‘sex’ or ‘age’ was included.

#### Bat flies

Both GLMM and GEE gave similar results and suggested the same biological conclusions: variation in infestation of bats with bat flies was associated with specific MHC class II *DRB* alleles, the inhabited roost, reproductive state and body size.

Analysing the impact of MHC *Noal-DRB* alleles independently of the ecological host characteristics, six different models had a ΔQAIC<2, identifying six different alleles as potentially influencing an individual's likelihood of being infected with bat flies. In all models, Noal-DRB*01, Noal-DRB*04, Noal-DRB*05 and Noal-DRB*10 were associated with lower and Noal-DRB*09 and Noal-DRB*11 with higher bat fly infestation (Table 1).

We combined ecological host characteristics with the allele information in further GLMM and GEE models. Both model approximations confirmed the relevance of the same ecological host characteristics, ‘roost’, ‘body size’, ‘reproductive state’ and the same MHC variables, namely Noal-DRB*04, Noal-DRB*09 and Noal-DRB*11, in explaining infestation of bats with bat flies (Table 3). Models including these alleles explained the variation better than a model neglecting these alleles (GEE: χ^2^ = 6.18–7.74., df = 2–3, p<0.05). Even so, GLMM models including these alleles in different combinations had low ΔQAIC-values (<0.69), a model without these alleles still explained the variation in the bats' infestation with bat flies reasonably well (ΔQAIC = 1.92). Alleles Noal-DRB*01, Noal-DRB*05 and Noa-lDRB*10 were found to be less important for the bats' infestation with bat flies in both GLMM (ΔQAIC: 2.32–4.65) and GEE (p>0.10). In models where *Noal-DRB* alleles were replaced by the variable ‘heterozygosity’, heterozygosity was validated not to influence the infestation with bat flies (GLMM with ‘heterozygosity’ ΔQAIC = 2.4; GEE: χ^2^ = 2.47, df = 1, p = 0.12). Of the ecological host characteristics, roost explained a substantial amount of the variation in infestation with bat flies (GLMM without ‘roost’: ΔQAIC = 59.8; GEE: χ^2^ = 73.8, df = 5, p<0.001). Bats of roost B, D and F showed a significantly higher infestation rate compared to animals from BCI (Table 2). Infestation intensity rose significantly with increasing ‘body size’ (GLMM without ‘body size’: ΔQAIC = 3.08; GEE: χ^2^ = 7.08, df = 1, p = 0.008). Also, reproductive state had a significant effect on infestation intensity (GLMM without ‘reproductive state’: ΔQAIC = 18.4; GEE: χ^2^ = 28.9, df = 4, p<0.001) with non-reproductive adult males being more parasitized than reproductive males. Also non-reproductive adult females were more infected than lactating and pregnant females (Table 3). Capture year also influenced the variation in bat fly infestation (GEE: χ^2^ = 34.9, df = 2, p<0.001), with animals sampled in 2007 showing a higher rate of infestation than animals sampled in 2006. Estimated parameters of the optimal GLMM model confirmed the results of the GEE and showed the same tendency (Table 3). ‘Sex’ had no effect on parasite load in models where sex was used instead of reproductive state (GLMM with ‘sex’: ΔQAIC = 4.24; GEE: χ^2^ = 3.38, df = 1, p = 0.07). The influence of ‘age’ on bat flies infestation varied: in GEEs age had no effect on infestation (χ^2^ = 0.22, df = 1, p = 0.64), but in GLMMs a model including age was best (ΔQAIC = 0), with subadults showing higher bat fly loads. However, a model without the variable age still was validated to be realistic (ΔQAIC = 2.02) indicating an uncertain effect of age on infestation. Estimates of all other covariates suggested a similar effect on infestation independently whether or not the variable ‘sex’ or ‘age’ was included.

**Table 3:** Bat fly infestation in *N. albiventris*.

GLMM			GEE			
Factors	Estimates ± SE	t-value	Factors	Estimates ± SE	Wald-Test	p-value
Intercept	−1.947±2.558	−0.76	Intercept	−0.899±1.039	0.75	0.386
Roost B^1^	0.089±0.631	0.14	Roost B^1^	0.674±0.283	5.65	0.017*
Roost C^1^	−0.106±0.592	−0.18	Roost C^1^	0.022±0.294	0.01	0.941
Roost D^1^	0.623±0.737	0.85	Roost D^1^	0.778±0.334	5.41	0.020*
Roost E^1^	−0.760±0.938	−0.67	Roost E^1^	0.056±0.389	0.02	0.886
Roost F^1^	3.427±1.105	3.11	Roost F^1^	2.077±0.354	34.5	<0.001***
Body size	0.283±0.228	1.25	Body size	0.295±0.110	7.08	0.008**
Female lactating^2^	−0.532±0.361	−1.48	Female lactating^2^	−0.531±0.188	7.59	0.005**
Female pregnant^2^	−0.675±0.566	−1.20	Female pregnant^2^	−0.489±0.215	5.14	0.023*
Male non-reproductive^3^	1.645±0.691	2.39	Male non-reproductive^3^	1.512±0.367	17.00	<0.001***
Noal-DRB*04	−0.162±0.249	−0.65	Noal-DRB*04	−0.164±0.117	1.97	0.161
Noal-DRB*09	0.276±0.260	1.06	Noal-DRB*09	0.207±0.154	1.81	0.178
Noal-DRB*11	0.364±0.389	0.96	Noal-DRB*11	0.379±0.166	5.23	0.022*
			Capture year 2007	0.605±0.238	6.15	0.009**
			Capture year 2008	−0.022±0.283	0.01	0.982

Estimated regression parameters and standard errors of combined ecological host characteristics and specific MHC class II *DRB* alleles on bat flies infestation obtained by GLMM (Laplace approximation with month and year as random effects) and GEE (with autocorrelation factor of roost: correlation parameter α = 0.08±0.07, overdispersion scale parameter = 2.8). Averaged estimates of GLMM models with ΔQAIC<2 are shown (QAIC_best_ = 135.0, overdispersion parameter of the full GLMM model = 3.34). N = 141.

1 compared to animals of BCI,

2 compared to non-reproductive adult females,

3 compared to reproductively active males.

### Comparison of MHC allele frequencies

We compared MHC-*DRB* allele frequencies of non-reproductive and reproductive adult males and subadults (Table S3) to investigate how alleles were transmitted into the next generation. Neither non-reproductive and reproductive males (*F*_ST_ = 0.006, p = 0.136 *G′*_ST_ = 0.055, D_est_ = 0.052; see Table S4 for confidence intervals) nor reproductive males and subadults (*F*_ST_ = 0.005, p = 0.243, *G′*_ST_ = 0.036, D_est_ = 0.033; Table S4) showed significant differences in their allele frequencies, but non-reproductive males differed in their allelic composition from subadults (*F*_ST_ = 0.025, p = 0.022, Bonferroni non-significant (α′≤0.016), *G′*_ST_ = 0.163, D_est_ = 0.152; Table S4).

**Figure 1:**
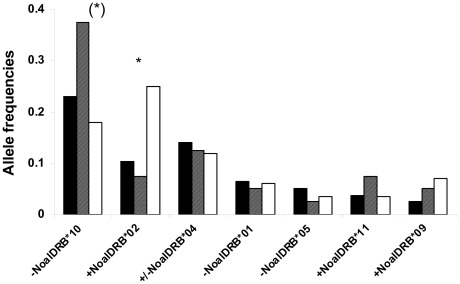
Allele frequencies of MHC class II *DRB* exon 2 influencing the ectoparasite infestation in *N. albiventris*. Distribution of *Noal-DRB* alleles influencing the ticks and bat flies infestation in reproductive males (black bars), subadults (grey bars) and non-reproductive males (white bars). ‘+’ indicates an association with an increased and ‘−’ with a decreased parasite load. Allele Noal-DRB*02 is significant accumulated in non-reproductive males and less frequent in subadults (χ^2^ = 10.07, p = 0.006, df = 2, Bonferroni significant, power (1-β err prob) 100%). Contrarily, allele Noal-DRB*10 is less frequent in non-reproductive males and accumulated in subadults (χ^2^ = 3.20, df = 1, p = 0.064, power 98%).

Further, we analysed differences in the allele distribution with respect to alleles which were identified to influence ectoparasite infestation (Noal-DRB*01, Noal-DRB*02, Noal-DRB*04, Noal-DRB*05, Noal-DRB*09, Noal-DRB*10, Noal-DRB*11; Table 1). Allele Noal-DRB*02 which was associated with a higher infestation rate of ticks, was more frequent in non-reproductive males than in reproductive males (χ^2^ = 6.95, df = 1, p = 0.008, power: 99%) and subadults (χ^2^ = 6.71, df = 1, p = 0.009, power: 98%) but no difference was found between reproductive males and subadults (χ^2^ = 0.223, df = 1, p = 0.43, power: 97%) (Figure 1). This effect was enhanced when the three groups were tested together (χ^2^ = 10.17, df = 2, p = 0.006, Bonferroni significant (α′≤0.016), power: 100%). Interestingly, Noal-DRB*10, the most frequent allele in the population (Table S3) which was associated with a decreased parasitism in ticks and bat flies showed an opposite pattern. Although not statistically significant, we found that Noal-DRB*10 tended to be less frequent in non-reproductive males and most frequent in subadults (χ^2^ = 3.20, df = 1, p = 0.064, power: 98%) (Figure 1). It occurred at an intermediate frequency in reproductive males. We found no difference, neither between reproductive and non-reproductive adult males (χ^2^ = 0.162, df = 1, p = 0.42, power: 99%) nor between reproductive males and subadults (χ^2^ = 1.63, df = 1, p = 0.16, power: 9%). All other alleles which occurred at minor frequencies in the study population (Table S3, Figure 1) were distributed without any significant differences among groups (χ^2^, results not shown).

## Discussion

We investigated the relationship between the individual immune gene constitution and ectoparasite loads in a free ranging population of *N. albiventris*. We analysed the impact of host characteristics like age, sex and reproductive state on ectoparasite infestation and we tested predictions of the ‘good-genes’ model, which postulates that males with well-adapted immune genes to coexisting parasites, have the ability to allocate more resources to reproduction. We found that in the neotropical bat *N. albiventris*, the infestation rate with ticks and bat flies was associated with various ecological traits, the reproductive status especially in males as well as with specific MHC class II *DRB* alleles.

### Environmental and ecological host characteristics associated with ectoparasite loads

All collected ectoparasite species have previously been reported for *N. albiventris* in Panama [41]. The tick species *O. hasei* is specific to bats and so far the sole tick species found to parasitize *N. albiventris* [32]. *Paradyschira* and *Noctiliostrebla* are bat fly genera that are specific to the bat genus *Noctilio* and may co-parasitize the same individual [58]. Our data are in line with investigations on ectoparasite assemblages in populations of *N. albiventris* in Paraguay and Venezuela, where a mean of 2.5 ectoparasites are reported to co-parasitize the same individual. Observed ectoparasite assemblages resembled our findings and included the tick *O. hasei*, a very common bat fly species of the genus *Paradyschiria* and a second less abundant fly of the genus *Noctiliostrebla*, which occurred on all bats also infested with the common fly [58]. During the study period the population has not been found to be faced with other severe diseases. Other ectoparasites like mites were observed only singularly and preliminary studies on intestinal helminth infestation showed that less than 12% of the population were infested by intestinal parasites. However, selection pressure on MHC class II alleles might also be caused by other external pathogens than the investigated ectoparasites.

Parasite abundance on bat hosts depends on complex interactions between environmental factors such as season and host characteristics such as roost usage, behaviour, body size, age, sex, reproductive state and individual immunocompetence [22], [24], [27], [59]–[61]. We found seasonal differences in ectoparasite loads on *N. albiventris*, both on a long- (years) and short-term scale (months). This may be caused by changes in environmental conditions as seasonal changes in temperature and humidity may cause fluctuations in the life cycle of ectoparasites [22] and thus may influence the infestation intensity of the host [26]. Since we cannot rule out the possibility that the observed seasonal differences are biased by unbalanced sampling, we controlled for random effects caused by capture year and month by using GLMMs.

Membership to a specific roost was identified to have an important impact on the infestation of bats with ectoparasites by both model approaches. This is not surprising, because roosts have been suggested as a primary source of ectoparasite transmission in bats [22], [28], [59], since ticks and bat flies depend on sheltered cavities for reproduction [30], [58].

In our *N. albiventris* population, infestation with ectoparasites was influenced by body size. The individual body size as a measure of a bat's surface area predicted increased prevalence of ticks and bat flies. Body size is thought to influence ectoparasite loads directly by limiting the available resources [62]–[65]. Across many host-parasite systems, male-biased parasitism has been postulated to be related to sexual size dimorphism [66]. Studies on bat hosts contrast this finding, with female bats being generally more heavily infested by ectoparasites [27], [67], [68]. We did not find any sex-biased differences in the infestation with bat flies in *N. albiventris*, and we found equivocal results concerning ticks. Females were validated only in one of the two model approaches (GLMM) to have higher tick loads than males, indicating also a minor effect of sex on tick burden.

Analysing the effect of individual reproductive state on parasite loads yielded intriguing results albeit sample sizes were quite low. Non-reproductive males were more heavily infested with both ectoparasites than reproductive males, suggesting a link between fitness and ectoparasite resistance. There is evidence that susceptibility to parasites might be related to the reproductive state also in female bats [24], [60], [69]. Females at different reproductive stages did not vary in their infestation rate with ticks. But, similar to that found in males, non-reproductive females showed higher infestation with bat flies than reproductive females (both, lactating and pregnant), indicating that individuals with better parasite resistance are more likely to reproduce. However, we did not detect early pregnancies, which refer to non-reproductive females and might have biased the results concerning ticks and bat flies. Variable results have been reported in other studies investigating infestation by ectoparasites of female bats at different reproductive stages [24], [60], [69]. These contrasting finding preclude general conclusions and indicate that complex processes act in specific bat-parasite systems.

Little is known about the roosting behaviour of *N. albiventris* in the wild and we cannot exclude that ectoparasite infestation might be influenced by roosting behaviour in relation to sex, age or even reproductive state. From captive individuals it is known that females and juveniles usually roost more closely to each other than do males (Dechmann, personal observation), which could explain the tendency of a higher ectoparasite load on females as well as subadults. Also age might influence an individual's susceptibility to parasites with an increased parasite load in juveniles and very old bats, most probably due to an ineffective immune system and self grooming capability [22]. According to our results a higher susceptibility to both ectoparasite taxa according to age was not unequivocally confirmed by the two modelling approaches applied and might be a result of the limited number of subadults investigated or may indicate a minor importance of age on ectoparasite loads. Unfortunately we do not know whether very old individuals were captured during the study, because once bats reach full size no field methods are available that include age determination of very old bats [34]. Long-term recapture studies would be necessary to ascertain this point.

### Impact of MHC class II DRB alleles on ectoparasite infestation

In addition to the complex ecological host characteristics and as a precondition to investigating the ‘good-genes’ model, we observed significant relationships between ectoparasite infestation and specific *Noal-DRB* alleles. We identified alleles associated with high (Noal-DRB*02, Noal-DRB*04, Noal-DRB*09, Noal-DRB*11) and low (Noal-DRB*01, Noal-DRB*04, Noal-DRB*05, Noal-DRB*10) ectoparasite abundance. Three of them had the same effect on both ectoparasite taxa (Noal-DRB*01, Noal-DRB*10, Noal-DRB*11), whereas others were associated either with tick or bat flies infestation (tick: Noal-DRB*02, bat flies: Noal-DRB*05 and Noal-DRB*09). Allele Noal-DRB*04 had a dual effect. It was correlated with an increased tick load and associated with a decreased bat fly infestation. It is known that although each MHC molecule has a high peptide binding specificity, it may accommodate several different peptides [70]. Moreover, resistance against one parasite can be conferred by multiple different MHC alleles [71]. Thus, co-evolutionary processes might not necessarily be entirely species specific [72].

During host parasite co-evolutionary processes, ectoparasites may also develop immunocompatibility with their hosts by sharing antigenic epitopes [30], [61]. Salivary proteins of ectoparasites are known to modulate host immunity in inhibiting regulatory as well as effector pathways involved in acquiring and expressing resistance [18], [61]. For example, antigen presenting macrophages and T_H_1 lymphocyte functions are suppressed by tick salivary gland extracts [17]. Shared antigenic epitopes between host and ectoparasites may explain the positive association of specific MHC molecules with specific ectoparasite taxa. Association of specific MHC class II *DRB* alleles with susceptibility to or protection against pathogens in mammals have been reported in numerous studies, including *DRB* alleles resistant to ectoparasites in cattle (*Bos taurus* [73]), white-tailed deer (*Odocoileus virginianus* [74]) and water vole (*Arvicola terrestris* [75]).

Besides direct responses of MHC mediated susceptibility to ectoparasites, the MHC may also contribute to individual attraction during the location of suitable hosts. Hosts are located by ectoparasites not only via respired carbon dioxide and body heat, but also through specific host odours [22]. Experimental analyses have shown that the individual body odour in vertebrates is influenced by immune genes of the MHC [76], [77]. This finding is supported by the fact that the MHC is in physical linkage with olfactory receptor genes in most vertebrates assessed so far [78]. Host odours are a particularly important cue for ectoparasites to differentiate among species [79], [80], and might also be used to differentiate between sexes and reproductive stages [24], [67]. Furthermore, there is evidence that odours produced from skin determine levels of attractiveness of human beings to mosquitoes [81], [82]. Verhulst and co-workers [82] found that the microbial community on the skin causes these differences in odorant cues. The authors hypothesize that the MHC may exert this influence of attractiveness by changing the skin microbiota composition and hence the volatiles produced by the bacteria and/or the human host. Our results could indicate that in *N. albiventris* specific *Noal-DRB* alleles might be responsible for attracting ectoparasite species to particular host individuals and may support the hypothesis of Verhulst and co-workers.

There is broad evidence that MHC mediated odours are used in mate selection with the consequence that reproduction among MHC dissimilar mates is favoured [83], [84] presumably to generate a genetically heterozygote offspring. Thus, there might be a reasonable possibility that beside direct selection through parasites also mating strategies might influences the MHC allele composition in the investigated *N. albiventris* population to some extend. However, an association between heterozygosity and ectoparasite load was only observed regarding tick infestation in one (GLMM) of the two model approaches. Against the predictions of the heterozygote advantage hypothesis [85] heterozygote individuals were associated with higher tick loads compared to homozygotes. We think that this equivocal result was caused by high frequency of Noal-DRB*02 but also Noal-DRB*11, which predominantly occurred in heterozygote individuals, both associated with increased tick burden.

The variation in the infestation with ectoparasites of a bat was best explained when ecological and genetic host characteristics were combined for analyses. Whereas ecological host characteristics showed a strong influence on the infestation the impact of immune genes were comparatively less powerful but still significant. Obviously ecological predictors which reflect the availability or exposure of ectoparasites, such as roost, season and available source (host body size) will be of overriding importance in the ectoparasite infestation compared to an individual's immune gene constitution or reproductive state with the latter acting both more on fine-scale parameters.

### Testing the ‘good-genes’ model based on MHC class II *DRB* constitution

To test the predictions of the ‘good genes’ model we focused on immunogenetic differences between non-reproductive and reproductive adult males to better understand the link between individual MHC class II *DRB* constitution, ectoparasite infestation and investment in reproduction. We additionally compared *DRB* variation between reproductive and non-reproductive males with that of subadults to detect indicators for selective reproductive success related to the immune gene constitution. Subadults differed in their overall *Noal-DRB* allele frequencies from non-reproductive, but not from reproductive males, possibly a consequence of the limited inheritance of alleles from non-reproductive males to subsequent generations. But using conventional F-statistics, statistical support disappeared after Bonferroni correction. However, Hedrick's *G′*_ST_ and Jost's D_est_ which are used to assess subtle genetic structuring in highly polymorphic loci such MHC showed higher support for an allelic differentiation between these groups. Non-reproductive and reproductive adult males differed at an intermediate level. We are aware that sample size limitations might have biased these results in both directions. However, these population differentiation tests are based on frequencies of all *Noal-DRB* alleles, including those alleles which were not relevant in the association to both ectoparasite taxa and might thus not precisely answer our question. When we analysed the distribution of specific alleles relevant for ectoparasite infestation, we obtained an unambiguous result concerning the two most frequent alleles in the population. Allele Noal-DRB*02, which was associated with a higher tick infestation, was significantly more frequent in non-reproductive males compared to reproductive males and was even less frequent in subadults. Furthermore, a noticeable, although non-significant accumulation of allele Noal-DRB*10, which was associated with low tick and bat fly loads, was observed at a high frequency in subadults but was less frequent in non-reproductive males.

Our results suggest that the MHC-*DRB* constitution contributes to the fitness of male bats as less infected individuals might have a higher reproductive success. Alleles which were common in strongly infected adult males were rare in subadults. This supports the assumptions of the ‘good-genes’ model: genetically well adapted males to prevailing parasites seem to be able to tolerate elevated costs of reproduction, whereas poorly adapted males suffer from increased parasite loads and seem not to be able to invest in the costly process of reproduction. Whether this holds true also for subsequent years requires ongoing investigations. It has been suggested by the immune competence handicap hypothesis [4] that testosterone might be the physiological mediator regulating the competition between reproductive investment and parasite defence in males. Genetic quality in terms of parasite resistance might modify this trade-off essentially [1], [4]. A potential association between MHC-types and testosterone production has been reported in white-tailed deer (*Odocoileus virginianus*), suggesting that males, carrying a certain MHC-type, bear the cost of elevated testosterone levels [86]. The relevance of the MHC for the reproductive effort in male fish has been demonstrated by Milinski and co-workers [87]. In male three-spined sticklebacks (*Gasterosteus aculeatus*) MHC dependent odour signals which are involved in mate choice are produced only when males are in reproductive state. The authors postulate that producing the MHC mediated olfactory signal is costly to the senders. Less healthy or parasitized males might thus stop producing this sexually selected trait or reduce their investment in reproduction when they can no longer afford to produce the costly signal. It remains to be investigated whether the susceptibility to ectoparasites in male *N. albiventris* increases during the mating period, and whether this has an effect on the investment in developed testes and the odour produced in the inguinal pockets for mating.

To conclude, our study indicates that besides the impact of ecological factors (e.g. roost, season), ectoparasite load is also influenced by MHC class II *DRB* allelic composition of an individual, shaping the trade-off between the cost of reproduction and immune defence. Thus, it provides evidence for the ‘good genes’ model based on immune gene variation which has rarely been investigated under natural conditions so far. Whether this also holds true for MHC class I in relation to virus infections will be the focus of ongoing studies.

## Supporting Information

Table S1 **Data collection of the** ***Noctilio albiventris*** **population in Panama in different roosts according to reproductive state.**
(DOC)

Table S2 **Data collection of the** ***Noctilio albiventris*** **population in Panama in different months and years according to reproductive state.**
(DOC)

Table S3 **MHC class II** ***DRB*** **exon 2 variability in the whole** ***N. albiventris*** **population, in non-reproductive adult males, reproductively active males and subadults.**
(DOC)

Table S4 **Test statistics on population differentiation using G′_ST_ (Hedrick 2005) and D_est_ (Jost 2008).**
(DOC)
