# Supplementary material for: Evidence for the ‘Good Genes’ Model: Association of MHC Class II DRB Alleles with Ectoparasitism and Reproductive State in the Neotropical Lesser Bulldog Bat, Noctilio albiventris
Source: PLoS One. 2012 May 16;7(5):e37101. doi: 10.1371/journal.pone.0037101 (PMC3353892; doi:10.1371/journal.pone.0037101)
Supplement: Table S1 — Data collection of the Noctilio albiventris population in Panama in different roosts according to reproductive state. (DOC) [file pone.0037101.s001.doc]

***Table S1.*** Data collection of the *N. albiventris* population in Panama in different roosts. Sample sizes according to reproductive state of the whole population (N = 214) are marked in bold, sample sizes of individuals with tick (N = 134) and bat flies (N = 166) data are given in parentheses.

| **Roost** | **Females** | | | **Males** | | **Subadults** |
| --- | --- | --- | --- | --- | --- | --- |
|  |  |  |  |  |  |  |
|  | **non-reproductive** | **lactating** | **pregnant** | **reproductive** | **non-reproductive** | **non-reproductive** |
| **BCI** | **4** (4, 1) | **0** | **3** (3, 3) | **13** (11, 8) | **8** (8, 4) | **1** (0, 0) |
| **A** | **10** (0, 5 ) | **16** (0, 5) | **0** | **10** (2, 7) | **8** (0, 2) | **9** (0, 0) |
| **B** | **30** (23, 30) | **14** (12, 14) | **8** (7, 7) | **3** (3, 3) | **16** (16, 16) | **3** (0, 3) |
| **C** | **8** (8, 8) | **1** (1, 1) | **4** (3, 4) | **3** (3, 3) | **10** (9, 10) | **1** (0, 1) |
| **D** | **8** (4, 8) | **2** (0, 2) | **0** | **3** (2, 3) | **1** (0, 1) | **6** (4, 6) |
| **E** | **3** (3, 3) | **0** | **0** | **1** (1, 1) | **0** | **0** |
| **F** | **0** | **0** | **0** | **7** (7, 7) | **0** | **0** |
