# Supplementary material for: Evidence for the ‘Good Genes’ Model: Association of MHC Class II DRB Alleles with Ectoparasitism and Reproductive State in the Neotropical Lesser Bulldog Bat, Noctilio albiventris
Source: PLoS One. 2012 May 16;7(5):e37101. doi: 10.1371/journal.pone.0037101 (PMC3353892; doi:10.1371/journal.pone.0037101)
Supplement: Table S2 — Data collection of the Noctilio albiventris population in Panama in different months and years according to reproductive state. (DOC) [file pone.0037101.s002.doc]

***Table S2.*** Data collection of the *N. albiventris* population in Panama in different months and years. Sample sizes according to reproductive state of the whole population (N = 214) are marked in bold, sample sizes of individuals with tick (N = 132) and bat flies (N = 166) data are given in parentheses.

| **Season** | **Females** | | | **Males** | | **Subadults** |
| --- | --- | --- | --- | --- | --- | --- |
|  |  |  |  |  |  |  |
|  | **non-reproductive** | **lactating** | **pregnant** | **reproductive** | **non-reproductive** | **non-reproductive** |
| **2006 _9** | **17** (0, 13) | **17** (0, 6) | **0** | **8** (0, 5) | **8** (0, 2) | **11** (0, 2) |
| **2007_10/11** | **3** (3, 3) | **2** (2, 2) | **0** | **9** (9, 8) | **1** (1, 1) | **0** |
| **2007_6** | **6** (3, 6) | **3** (0, 3) | **0** | **1** (1, 1) | **1** (0, 1) | **7** (4, 7) |
| **2007_9** | **13** (11, 13) | **9** (9, 9) | **0** | **6** (6, 6) | **14** (13,14 ) | **0** |
| **2008_2/3** | **11** (11, 11) | **2** (2, 2) | **11** (9, 10) | **1** (1, 1) | **3** (3, 3) | **0** |
| **2008_4/5** | **3** (2, 2) | **0** | **1** (1, 1) | **1**(1,1) | **4** (4, 1) | **0** |
| **2008_9** | **0** | **0** | **0** | **2** (2, 2) | **0** | **0** |
| **2008_10/11** | **10** (9, 8) | **0** | **3** (3, 3) | **12** (9, 8) | **12** (12, 10) | **2** (1, 1) |
