# Supplementary material for: Evidence for the ‘Good Genes’ Model: Association of MHC Class II DRB Alleles with Ectoparasitism and Reproductive State in the Neotropical Lesser Bulldog Bat, Noctilio albiventris
Source: PLoS One. 2012 May 16;7(5):e37101. doi: 10.1371/journal.pone.0037101 (PMC3353892; doi:10.1371/journal.pone.0037101)
Supplement: Table S3 — MHC class II DRB exon 2 variability in the whole N. albiventris population, in non-reproductive adult males, reproductively active males and subadults. (DOC) [file pone.0037101.s003.doc]

***Table S3.*** MHC class II *DRB* exon 2 variability in the whole *N. albiventris* population, and in non-reproductively adult males, in reproductively active males and in subadults. Sample size (N), number of alleles (No) and allele frequencies are shown.

|  |  | **All** |  | **Males**  **non-repro** | **Males**  **repro** | **Subadults** |
| --- | --- | --- | --- | --- | --- | --- |
| **N** |  | 214 |  | 42 | 40 | 20 |
| **No** |  | 18 |  | 14 | 15 | 12 |
| ***NoalDRB**01** |  | 0.040 |  | 0.060 | 0.064 | 0.050 |
| ***NoalDRB**02** |  | 0.175 |  | 0.250 | 0.103 | 0.075 |
| ***NoalDRB**03** |  | 0.009 |  | - | 0.013 | - |
| ***NoalDRB**04** |  | 0.117 |  | 0.119 | 0.141 | 0.125 |
| ***NoalDRB**05** |  | 0.044 |  | 0.036 | 0.051 | 0.025 |
| ***NoalDRB**06** |  | 0.040 |  | 0.036 | 0.026 | 0.025 |
| ***NoalDRB**07** |  | 0.005 |  | 0.012 | - | - |
| ***NoalDRB**08** |  | 0.042 |  | 0.071 | 0.038 | 0.100 |
| ***NoalDRB**09** |  | 0.054 |  | 0.071 | 0.026 | 0.050 |
| ***NoalDRB**10** |  | 0.255 |  | 0.179 | 0.231 | 0.375 |
| ***NoalDRB**11** |  | 0.028 |  | 0.036 | 0.038 | 0.075 |
| ***NoalDRB**12** |  | 0.088 |  | 0.071 | 0.128 | 0.025 |
| ***NoalDRB**13** |  | 0.028 |  | 0.024 | 0.026 | 0.025 |
| ***NoalDRB**14** |  | 0.005 |  | - | 0.013 | - |
| ***NoalDRB**15** |  | 0.005 |  | - | - | - |
| ***NoalDRB**16** |  | 0.007 |  | - | - | - |
| ***NoalDRB**17** |  | 0.044 |  | 0.024 | 0.038 | 0.05 |
| ***NoalDRB**18** |  | 0.014 |  | 0.012 | 0.038 | - |
