# Supplementary material for: Evidence for the ‘Good Genes’ Model: Association of MHC Class II DRB Alleles with Ectoparasitism and Reproductive State in the Neotropical Lesser Bulldog Bat, Noctilio albiventris
Source: PLoS One. 2012 May 16;7(5):e37101. doi: 10.1371/journal.pone.0037101 (PMC3353892; doi:10.1371/journal.pone.0037101)
Supplement: Table S4 — Test statistics on population differentiation using G′ST (Hedrick 2005) and Dest (Jost 2008). (DOC) [file pone.0037101.s004.doc]

***Table S4.*** Test statistics on population differentiation using G’_ST_ (Hedrick 2005) and D_est_ (Jost 2008).

|  | **Groups compared** | **Df** | **Diversity**  **Parameter** | **Pairwise Distance** | **Bootstrap**  **Estimate** | **Variance** | **Std Err** | **Confidence Interval** |
| --- | --- | --- | --- | --- | --- | --- | --- | --- |
|  |  |  |  |  |  |  |  |  |
|  | **Males non-repro** | 2 | G’_ST_ |  | 0.178 | 0.006 | 0.002 | 0.052-0.326 |
|  | **Males repro** |  | D_est_ |  | 0.162 | 0.005 | 0.002 | 0.048-0.299 |
|  | **Subadults** |  |  |  |  |  |  |  |
|  |  |  |  |  |  |  |  |  |
|  |  |  |  |  |  |  |  |  |
|  | **Males non-repro** | 1 | G’_ST_ | 0.055 | 0.130 | 0.007 | 0.003 | 0.003-0.287 |
|  | **Males repro** |  | D_est_ | 0.052 | 0.123 | 0.006 | 0.003 | 0.003-0.171 |
|  |  |  |  |  |  |  |  |  |
|  | **Males non-repro** | 1 | G’_ST_ | 0.036 | 0.234 | 0.013 | 0.004 | 0.042-0.045 |
|  | **Subadults** |  | D_est_ | 0.033 | 0.219 | 0.012 | 0.003 | 0.040-0.423 |
|  |  |  |  |  |  |  |  |  |
|  |  |  |  |  |  |  |  |  |
|  | **Males repro** | 1 | G’_ST_ | 0.163 | 0.133 | 0.010 | 0.003 | -0.017-0.327 |
|  | **Subadults** |  | D_est_ | 0.152 | 1.123 | 0.008 | 0.003 | -0.015-0.037 |
|  |  |  |  |  |  |  |  |  |

Df: degrees of freedom, D_est_: Jost’s diversity index (Jost 2008), G’_ST_: Hedrick’s diversity index (Hedrick 2005), Std Err: Standard error
